# A Glimpse into Chromatin Organization and Nuclear Lamina Contribution in Neuronal Differentiation

**DOI:** 10.3390/genes14051046

**Published:** 2023-05-06

**Authors:** Salvatore Martino, Pietro Salvatore Carollo, Viviana Barra

**Affiliations:** 1 Department of Biological Chemical and Pharmaceutical Sciences and Technologies, University of Palermo, 90128 Palermo, Italy; salvatore.martino01@unipa.it; 2 Institute of Molecular Bioimaging and Physiology, National Research Council (IBFM-CNR), 90015 Cefalù, Italy

**Keywords:** chromatin organization, nuclear lamina, epigenetics, neuronal differentiation

## Abstract

During embryonic development, stem cells undergo the differentiation process so that they can specialize for different functions within the organism. Complex programs of gene transcription are crucial for this process to happen. Epigenetic modifications and the architecture of chromatin in the nucleus, through the formation of specific regions of active as well as inactive chromatin, allow the coordinated regulation of the genes for each cell fate. In this mini-review, we discuss the current knowledge regarding the regulation of three-dimensional chromatin structure during neuronal differentiation. We also focus on the role the nuclear lamina plays in neurogenesis to ensure the tethering of the chromatin to the nuclear envelope.

## 1. Introduction

The DNA sequence of all cells in the body is the same even though the cells do not play the same function. Each cell type is characterized by a precise orchestration of gene expression for the production of all proteins necessary for specific cellular function. Hence, not all genes are active in all cells at any given time, and their expression is tightly regulated by the epigenome. The Greek preposition “*epi*” means on or above, and “epigenome” takes into account all those chemical modifications “on” the DNA molecule and histone proteins without affecting DNA sequence. The epigenome acts as a film director, assigning different roles to each cell type by defining when and what genes are accessible to transcription factors to regulate their expression. Additionally, the epigenome contributes to gene expression through the modification of the three-dimensional organization of the genetic material (chromatin). The epigenome regulates the DNA via DNA methylation on the cytosine of CpG dinucleotides and post-translational modifications of the histone proteins, but also via the activity of non-coding RNA and chromatin remodellers [1]. The effects and the meaning of these modifications are diverse, and many of them are still the subject of investigation. We will consider those modifications known to be involved in the spatial rearrangement of DNA and regulation of gene expression. In this regard, DNA methylation is involved in different aspects of genome regulation such as DNA compaction [2,3] and gene regulation [4]. Indeed, methylation leads to DNA compaction, establishing the repression of both repetitive elements and genes if it occurs at gene promoters [5,6]. DNA methylation can also allow or block the DNA binding of transcription factors and other proteins of the epigenetic families depending on different genomic context, thus affecting the activities of transcriptional factors [7,8]. Histone acetylation has been generally correlated with gene activity, in contrast to histone deacetylation, which has been linked to transcriptional repression. The methylation of histones instead can lead to different effects depending on the lysine residue that is modified and its location within the gene (see Section 2, ‘Three-Dimensional Organization of Chromatin within the Nucleus’, for further details) [9,10]. Importantly, histone modifications can also recruit other proteins of the epigenetic families, such as chromatin remodellers and proteins with the chromodomain or bromodomain that allow the binding of methylated and acetylated histones, respectively, to further regulate chromatin structure [11,12]. Chromatin remodellers belong to a family of ATPase proteins and use the energy derived from the hydrolysis of ATP to modulate the contacts between histones and DNA in the nucleosome. With this ability, remodellers can expel or slide nucleosomes, and remove and replace histone dimers to regulate the access to DNA [13]. Members of the SWI/SNF family, for example, generally lead to an increase in chromatin accessibility through sliding and ejecting nucleosomes [14]. On the other hand, chromatin remodelling complexes belonging to the ISWI family, by facilitating nucleosome assembly and spacing, can both activate and repress transcription depending on the gene context [15].

Long non-coding RNAs (lncRNAs) can modulate chromatin structure and regulate the transcription of adjacent and distant genes, but can also affect RNA splicing, stability and translation [16,17]. 

Within the nucleus, chromatin is organized in nuclear topological domains that differ depending on the cell type and stage of differentiation. The nuclear spatial organization, or nuclear architecture, ensures the correct performance of the transcriptional programs within cells, thus giving it an important role in cellular differentiation and development. Furthermore, nuclear architecture is dictated not only by the epigenome but also by the nuclear lamina, which both physically supports the nucleus and anchors chromatin to the nuclear envelope [18]. This suggests that the nuclear lamina could also be involved in the establishment of the cell transcriptional programs. Interestingly, alterations in nuclear lamina components per se result in gene expression changes. Mouse fibroblasts lacking full-length Lamin B1 (see Section 3, ‘Nuclear Envelope: LINC Complex and Nuclear Lamins’) showed alterations in gene expression, with many genes (834) being down-regulated and a few (129) up-regulated, suggesting a role in both the repression and activation of transcription through the repositioning of chromosomes in interphase [19]. Lamina-dependent changes in the gene expression program have been shown to affect cell fate commitment. In this context, it has recently been observed that the Lamin A heterozygous mutation *LMNA* T10I in human-induced pluripotent stem cell-derived cardiomyocytes (hiPSC-CMs) causes increased expression of alternative fate genes (i.e., neurogenesis, epithelium development…), which are normally repressed as bound to the nuclear lamina [20]. Moreover, lamina components can also regulate transcription indirectly during cell differentiation. As such, Lamin B1 controls the expression of oxidative stress genes by sequestering the Oct-1 factor. *Malhas* and colleagues demonstrated that depletion of the C-terminus of Lamin B1 causes increased susceptibility to ROS formation in a model of mouse embryonic fibroblasts (Lmnb1^Δ/Δ^ cells) [21]. It has been shown that gene transcription can also be regulated by the nuclear lamina through the process of “mechanotransduction”—a process cells use to convert physical cues, via the cytoskeleton, into biochemical signals [22]. A direct involvement of mechanical stimuli on animal differentiation has been observed. In fact, by applying a constraint on Oregon R *Drosophila Melanogaster* embryos, the *Twist* factor, important for *Drosophila* development, increased both in terms of mRNA and protein due to the nuclear accumulation of Armadillo protein [23]. In addition to constraints, substrate stiffness can also be a discriminant factor towards fate development. In fact, *Engler* and colleagues demonstrated that human mesenchymal stem cells (MSCs) differentiated towards the brain, muscle or bones if these cells were plated onto a substrate whose stiffness resembled the one found in the brain (0.1–1 kPa), muscle (8–17 kPa) or bone (25–40 kPa), respectively. In addition, the researchers found that this lineage commitment was abrogated by the inhibition of the motor protein non-muscle myosin II (NMM II) by treating cells with the cytoskeletal drug blebbistatin [24]. Thus, it is clear that force sensing with its timely and proper transmission can dramatically influence and change development and differentiation as well. 

In this review, we will focus on the current knowledge about the role played by chromatin organization and nuclear lamina in mammals’ neurodevelopment. We will first give an overview of the three-dimensional organization of chromatin and the nuclear envelope structure. Afterwards, we will give a general introduction about the developmental process of the nervous system. We will then discuss examples showing the importance of chromatin organization and nuclear lamina in the development and differentiation of neuronal cells. 

## 2. Three-Dimensional Organization of Chromatin within the Nucleus

The three-dimensional organization of chromatin within the nucleus is critical for the regulation of gene expression and cellular function. In eukaryotic cells, the DNA is organized with different levels of compaction to fit within the micron-sized nuclear space. The DNA wraps around a complex of eight histone proteins constituting the nucleosomes, resulting in the 10 nm fibre known as chromatin that can reorganize into a 30 nm fibre, at least in vitro; however, chromatin is mainly found as a disorganized structure, heterogeneous and diverse in diameter and with a high bendability [25]. Indeed, chromatin can organize itself into loops due to the presence of architectural proteins [26,27]. Different models have been proposed. The first one takes into account the insulator proteins, DNA-binding proteins that, through their ability to interact with each other, can bring together distant genomic sites, allowing DNA element interactions (i.e., enhancer–promoter interactions) only inside the loop domain (isolation). As shown in *Drosophila*, insulators can interact with each other at high distances, and this interaction can either stabilize or suppress the enhancer–promoter communication depending on the orientation of the enhancer and promoter relative to the insulator elements [28]. However, this model does not seem to be highly accurate in mammals. Indeed, some authors, by combining Hi-C data and a novel mathematical theorem, showed that chromatin loops are formed according to the model of chromatin extrusion. In such a model, the insulator protein complex (i.e., cohesin or CTCF) initially binds random loci in the chromatin fibre, forming a short loop. Then, the two subunits of the complex, by moving in opposite directions along the fibre, elongate the loop till they meet a properly oriented insulator element, and the extruded chromatin forms a domain [29]. This model has been renamed as the “tethered inchworm model”, where the SMC cohesin complex uses an ATP-dependent ability to provide the motor force that enlarges the chromatin loop [30]. This model has been proposed as the mechanism that induces the reorganization of chromatin and that underlies the formation of the “topologically associating domains” (TADs), domains of chromatin, at the sub-megabase scale level, where genome regions display a high frequency of interaction [31,32,33]. To support chromatin interactions, different players are involved. In this context, high-resolution maps were generated to study the genome architecture across seven genomic loci in embryonic stem cells (ESCs) and neural progenitor cells (NPCs). These showed that chromatin interactions are enabled by the presence of insulator/architect proteins, such as CTCF or cohesins described above, which anchor long-range constitutive interactions [34]. Other short-range enhancer–promoter interactions within and between larger chromatin subdomains were also revealed to be induced by Mediator, a transcriptional coactivator in complex with cohesin [34]. Chromatin looping inside TADs has indeed been shown to be correlated with transcription regulation. In mice, regulatory domains were able to activate the LacZ reporter gene driven by a weak promoter within the same TAD irrespective of the distance, suggesting that TADs are a functional subunit of the genome [35]. With regard to this, it has been recently shown through the 5C technique that intra-TAD interactions happen at a high frequency. Inter-TAD interactions can also happen, though at a low rate, at a genomic distance greater than 200 kb [32,36]. In fact, the presence of TAD boundaries, enriched for the binding of architectural proteins (mainly CTCF in mammals), restrains the interactions between regulatory sequences (i.e., enhancer, silencer) and target genes of different TADs [31,32,37,38]. In this way, TADs greatly contribute to gene expression regulation. In interphase nuclei, chromosomes form chromosomal territories [39], with TADs being subunits of them [40]. Within chromosomal territories, two types of compartments can be identified. The “A” compartment is the more relaxed configuration of chromatin fibre called euchromatin, which is usually actively transcribed and mostly found in the interior of the nucleus, whereas the “B” compartment is the more compact chromatin called heterochromatin, which is mainly transcriptionally repressed and found preferentially at the nuclear periphery and in the nucleolus [18,41,42] (details of eu- and heterochromatin will be discussed below in this paragraph). In this regard, it has been observed that some specific TADs are associated with the nuclear lamina (see Section 3, ‘Nuclear Envelope: LINC Complex and Nuclear Lamins), an intermediate filament network lining the inner surface of the nuclear envelope. This association forms the so-called “lamina-associated domains” (LADs) with a repressive role in gene expression [43]. 

From the epigenetic point of view, chromatin compartmentalization and the degree of compaction are determined by specific modifications of both histones and DNA. The relaxed configuration of euchromatin is accompanied by histone acetylation, which neutralizes the positive charge of lysine residues, favouring chromatin opening [10]. Additionally, transcriptionally active genes are usually marked by H3K4 methylation [44]. On the other hand, condensed chromatin is induced by the deacetylation of histones, DNA methylation and methylation of specific residues of histones. Characteristic markers of heterochromatin are trimethylated H3K9, trimethylated H3K27 and trimethylated H4K20 [5,18].

## 3. Nuclear Envelope: LINC Complex and Nuclear Lamins

The nuclear envelope (NE) is made up of the outer nuclear membrane (ONM) and the inner nuclear membrane (INM) and acts as a physical barrier that separates the cell nucleus, harbouring chromatin, from the cell cytoplasm [18]. Between the INM and the ONM lies the perinuclear space (PNS), with a width of 30–50 nm [45], and juxtaposed to the nucleoplasmic side of the INM, there is the nuclear lamina.

The LINC complex (linker of nucleoskeleton and cytoskeleton) spans the INM and ONM and provides mechanical coupling between the actin cytoskeleton and the nucleus [46,47]. This is possible thanks to both the ONM and the INM components of the LINC complex, the nesprins (nuclear envelope spectrin repeat proteins) and the SUN proteins (Sad1p and UNC-84 homology), respectively [48,49,50]. Specifically, nesprin proteins act as a bridge between the cell cytoskeleton and the INM by binding actin on one side and SUN proteins on the other side, whereas SUN proteins interact on the nucleoplasmic side with either the nesprins in the PNS or the nuclear lamins underneath the INM, thus indirectly connecting cell cytoskeleton with the nuclear lamina [45]. 

In humans, three genes encode the components of the nuclear lamina, *LMNA*, *LMNB1* and *LMNB2*. The products of the *LMNA* gene are lamins A and C, which are translated following an alternative splicing event on exon 10. The *LMNB1* and *LMNB2* genes encode Lamin B1 and Lamin B2, respectively [45]. Lamin B1 is bound by LBR (Lamin B receptor), which, in turn, interacts with MeCP2 (Methyl-CpG binding protein 2) and HP1α (heterochromatin protein 1), which are responsible for the binding of 5-Methylcytosine and H3K9me3 on the DNA, respectively, both epigenetic markers of heterochromatin [18,51,52].

The elaborate organization of the nuclear envelope provides structural support to the nucleus and allows the tethering of heterochromatin domains to the nuclear periphery, supporting genome compartmentalization (see Section 2, ‘Three-Dimensional Organization of Chromatin within the Nucleus’). As a result, alterations in lamina components jeopardize nuclear structure. With regard to this, a lack of Lamin B1 has been associated with nuclear bleb formation [53,54]. Interestingly, nuclear blebbing can also result from an increase in euchromatin or a decrease in heterochromatin without the perturbation of lamina components, demonstrating that chromatin state has a role, together with nuclear lamina, in the preservation of the structure of the nucleus [54]. 

The organization of the nuclear envelope also ensures that every mechanical cue perceived by the cells is transmitted, via the cytoskeleton and the nuclear envelope, to the nuclear interior, thus regulating chromatin dynamics and, as a consequence, gene transcriptional events [55,56,57]. In fact, it has been demonstrated that LINC complex ablation, via the dominant negative form of Nesprin 2G (DNKASH), caused impaired force transmission from the cell cytoskeleton to the nucleus in a microneedle manipulation assay as well as impaired wound closure in a wound healing assay in mouse embryonic fibroblasts (MEFs) [58]. In addition, LINC complex abrogation altered the expression of genes related to both cytoskeleton and focal adhesions as well as to the nuclear envelope in NIH 3T3 fibroblasts [59]. 

The nuclear envelope is also involved in transcriptional regulation independently of mechano-sensing, through the nuclear lamina, as mentioned in the introduction, which is able to bind the epigenetic markers of heterochromatin. The involvement of the nuclear lamina in gene regulation during neuronal differentiation will be discussed in Section 6, Implication of Nuclear Lamina in Neuronal Development.

## 4. Neurogenesis: An Overview

During embryogenesis, at the beginning of gastrulation, human embryonic stem cells from the ectoderm start to give rise to human neural progenitor cells (hNPCs) characterized by a radial alignment and a bipolar morphology. These cells undergo symmetric divisions for self-renewal to increase the size of the cell pool, which, by the end of gastrulation, forms the *neuronal plate* along the rostral–caudal midline of the upper layer of the embryo. The ridges of the neural plate then fold inward to create the neural tube [60]. At this point, the hNPCs, depending on their position, will differentiate into either neurons or glia to construct the nervous system. The rostral region of the neural tube will give rise to the brain, while the caudal region will give rise to the hindbrain and spinal column [60]. In the process mentioned above, in addition to the members of the TGF-β family (which have different roles, from maintaining the pluripotency of embryonic stem cells to mesenchymal differentiation), both chromatin organization and epigenome are crucial players. In this review, we have decided not to tackle the issue of epigenetic modifications characterizing neuronal development that has been extensively reviewed recently [61,62]. Instead, we have focused on the chromatin structure changes and their relationship with the nuclear lamina during neuronal development.

## 5. Chromatin Structure Involvement in Neural Development

Epigenetic modifications are usually associated with the regulation of the development and differentiation processes. However, the different compartmentalization of chromatin also plays an important role in these contexts. Studies on murine embryonic stem cells (mESCs) as well as on human embryonic stem cells (hESCs) revealed, through transmission electron microscopy, that undifferentiated ESCs have euchromatin-rich nuclei with prominent nucleoli, whereas differentiating ESCs are characterized by increasingly condensed heterochromatin distributed in a diffuse granular pattern and as a dense strip beneath the nuclear edge [63]. Similarly, in *Drosophila*, DamID mapping of LADs showed that differentiated neurons have enhanced, HP1α-rich heterochromatin associated with the nuclear lamina. In contrast, Kc167 cells, of embryonic origin, present LADs lacking HP1α [64]. These changes are accompanied by specific histone epigenetic modifications, mainly the acetylation and methylation of lysine 9 of histone H3, which are important in determining the developmentally regulated genes as being active euchromatin or repressed heterochromatin. This is a fundamental step towards cell fate commitment during development. 

In this regard, the analysis of the three-dimensional organization of the genome during neuronal murine development showed that mESCs are characterized by open chromatin with epigenetic marks of active gene expression (i.e., high levels of H3/H4 acetylation and trimethyl H3K4, or low levels of histone trimethylation on lysine 27), and few compacted chromatin domains. Instead, murine neural progenitor cells (mNPCs) have condensed chromatin with more heterochromatic domains clustered in chromocenters, bright DAPI-positive domains of constitutive heterochromatin [65,66,67,68]. *Nakao*’s group also showed that even mESCs are characterized by chromocenters that are smaller than mNPCs and integrated into larger foci in post-mitotic neurons (mPMNs) [66]. The number and shape of chromocenters also change during the differentiation of the neural cell types [69,70,71,72]. Specifically, chromocenter numbers decrease in murine Purkinje cells from the day of birth till postnatal day 6 and then increase till mice become adults [70]. Additionally, the deposition of epigenetic markers is involved, such as the active histone mark trimethylated lysine 4 of histone H3, which increases at chromocenters during neuronal differentiation in the neocortex. This is accompanied by a parallel increase in the transcription of major satellites [73]. In accordance with changes in the chromatin state (open/closed), the interior of the TADs changes. However, the boundaries of the TADs stay invariant during development [74]. Interestingly, by inducing mESCs to become neuronal committed cells (mNCCs) with retinoic acid, *Stachowiak*’s group identified the TADs in mESCs and mNCCs, showing the relocation of the position of TAD boundaries in mNCCs compared to the mESCs [75]. Specifically, mNCCs increase the expression of nuclear fibroblast growth factor receptor 1 (nFGFR1) [75], which strongly correlates with neuronal differentiation by regulating pluripotency genes [76]. In addition, nFGFR1 also works as a protein insulator, leading to a reorganization of the chromatin loops and TADs [75]. Finally, CTCF insulator was reduced in comparison to the mESCs [75]. From these studies, the reorganization of TADs has been found to be related to not only the loss of stem potency but also to cell differentiation. Similar events characterize neuronal development in *Drosophila*. In vivo cell-type-specific chromatin maps revealed that in neural stem cells (NSCs), the stemness genes, involved in the establishment of NSC identity, and cell cycle genes were found within permissive chromatin marked by H3K27ac, whereas during the differentiation of neurons, they move to a repressed HP1-rich chromatin. In contrast, most pro-neuronal genes are located within TrxG-permissive chromatin in neurons, while in NSCs, they are found in the silent “black” chromatin (lacking epigenetic modifications) or trithorax group (TrxG)-repressed chromatin (containing both H3K27ac and the linker histone H1) [77].

## 6. Implication of Nuclear Lamina in Neuronal Development

The redistribution of chromatin is needed during development to specify the cell type’s fate and is essential to cell fitness and function. Alterations in key components of the chromatin 3D reorganization such as the nuclear lamina induce aberrations during development that potentially lead to organism death [78]. Indeed, a homozygous *LMNA* mutation leads to prenatal lethality in humans, whereas in mice, *LMNA* mutation is not lethal and results in different pathologies a few weeks after birth [78]. Intriguingly, upon differentiation, some genes move towards or away from the nuclear lamina [79] according to their activation or repression state. For example, mNPCs, after induction from mESCs, showed relocation of the pluripotency genes towards the nuclear lamina, a position that is maintained even after further differentiation [80]. *Van Steensel*’s group analysed mNPC differentiation and discovered an increased interaction between the nuclear lamina and 633 genes, some of which are “stemness” genes, i.e., *Nanog*, *Klf4* and *Oct4* [80]. Since these genes move to the nuclear periphery, they are usually associated with the LADs, implying a heterochromatinization during differentiation [43,81]. *Williams’* group analysed the positioning of Mash1, a proneuronal factor, and noticed that the *Mash1* locus is mostly located at the nuclear periphery in ESCs, which, upon neuronal induction, relocates to the interior part of the nucleus. Moreover, this study demonstrates that repositioning is directed in a cell-type-specific manner. Indeed, other differentiated cell types were characterized by *Mash1* located at the nuclear periphery, similar to the ESCs [82]. However, it is important to mention that relocation is not necessarily equivalent to gene activation since the transcription of some neuronal genes is associated with further differentiation [80]. This is the case for the brain *Pcdh9* gene [80] and several neuronal genes that dissociate from the nuclear lamina even if they are not actively transcribed in mNPCs. 

Considering the strong interdependence between chromatin organization, heterochromatin and nuclear lamina, as discussed above and in *Carollo* and *Barra 2023* [18], it is not surprising that nuclear lamina and its mechanics have been shown to be important for neuronal development, as demonstrated by the elegant works conducted by *Young*’s group [83,84]. In 2011, *Coffinier* and colleagues demonstrated that the deficiency of Lamin B1 (Lmnb1Δ/Δ) causes problems in the development of the cerebral cortex in mouse embryos, with impairments in neuronal migration as well [83]. This was accompanied by a reduction in neuronal progenitor cells and an increase in apoptotic cell death. Moreover, Lamin B1 deficiency was the cause of misshapen cell nuclei of cortical neurons, which has been correlated with the alteration of the heterochromatin:euchromatin ratio in other cell contexts [18]. In addition, KO of either Lamin B1 or Lamin B2 via Cre recombinase in mouse embryos caused both reduced cranium and cerebral cortex size. The cortex was also smaller and showed atrophy in double-knockout Lamin B1 and Lamin B2 mice compared to the single-KO condition. Moreover, adult mice lacking either Lamin B1 or Lamin B2 exhibited problems in the layering of cortical neurons, as demonstrated by the absence of Cux1, a marker of layer II/III, in most of the neurons. Lamin B1 KO neurons displayed nuclear blebs (one bleb/nucleus) with an asymmetric distribution of Lamin B2 [83]. Atypical nuclei can also be formed when Lamin B1 does not correctly localize in the nuclear envelope, which is the case of mouse mutants for Lamin B1 that cannot be farnesylated (Lmnb1CS/CS). Indeed, *Jung* and colleagues demonstrated that the mutated Lamin B1 mislocalises in the nucleus in a honeycomb fashion, which correlates with strong defects in cell nucleus shape. Specifically, it has been observed that during in vitro migration, the NPCs of Lmnb1CS/CS mice have dumbbell-shaped nuclei and blebs. In these cells, Lamin B1 was mainly at the leading edge (towards the direction of the migration). Strikingly, the opposite side of the cell (the trailing edge) was occupied by the bulk of chromatin, called “naked chromatin” because it is disconnected from the nuclear lamina [84]. The authors supposed that the dumbbell-shaped nuclei form because of a weakened interaction between the nuclear lamina and the inner nuclear membrane due to the mutant Lamin B1. In detail, during neuronal migration, the nuclear lamina follows the nucleokinesis and is pulled forward by the microtubule’s cytoskeleton, but it loses connection with the trailing edge of the nucleus because of Lmnb1CS. Consequently, chromatin is not trapped in the nuclear lamina meshwork and eventually escapes through the honeycomb-like pores remaining in the trailing edge, uncoupling it from the nuclear lamina. However, it is also possible that other mechanisms are involved in this event. For example, the chromatin could not be affixed anymore to the nuclear lamina due to mutant Lamin B1. We should keep in mind that Lamin B1 connects with chromatin and binds LBR, which, in turn, tethers heterochromatin to the inner nuclear membrane [72,85].

All the above strongly suggests that Lamin B1 is essential in retaining chromatin bound to the nuclear lamina. Indeed, it has been shown that Lamin B1 is important for TAD–TAD interaction in an mESC model of TKO for *LMNA*, *LMNB1* and *LMNB2* and that, more specifically, Lamin B1 depletion causes LAD detachment from nuclear lamina with a subsequent impact on chromatin redistribution and, thus, chromatin dynamics in MDB-MB-231 breast cancer cells [86,87]. Intuitively, this can affect the three-dimensional organization of chromatin, which strongly correlates with the gene expression program as discussed above. This can be evidenced by the fact that Lmnb1CS/CS mice have severe neurodevelopmental abnormalities with the formation of a flattened cranium and reduced size of the brain [84]. Moreover, *Gigante* and colleagues have recently shown that conditional depletion of Lamin B1 in postnatal, quiescent mouse stem cells negatively impacts their commitment towards olfactory sensory neurons, with down-regulation of genes implied in the response to odour stimuli [88]. In addition, the presence of a functional Lamin B1 is required to ensure genome integrity and cell viability during neuronal migrations for the development of both the cerebral cortex and retina [89,90].

## 7. Conclusions

**Figure 1:**
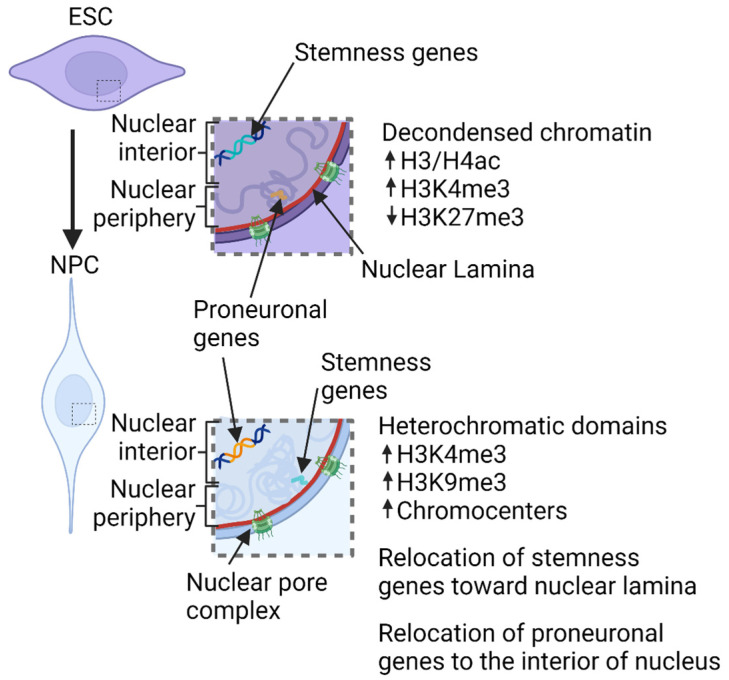
Schematic summarizing the events that, by regulating chromatin three-dimensional organization, ensure a faithful neuronal differentiation. Created with BioRender.com (accessed on 1 May 2023).

Development is a complex event, with many aspects of cell regulation involved. For instance, changes in the cell microenvironment can result in modifications of the cell’s phenotype, contributing to the determination of cell fate. Nevertheless, the manual of cell differentiation is written on the DNA and, thus, the plasticity of cells depends on its regulation. DNA regulation is, in fact, an intricate and intriguing event in a cell’s life. Epigenetic factors, cis-acting elements (insulators), non-coding RNAs and DNA compaction are important aspects of DNA regulation. The 3D organization of chromatin and the positioning of the genes seem to be involved in the regulation as well, having a role in the determination of gene transcription patterns (Figure 1). Here, we focused on the importance of the 3D organization of chromatin and the epigenetic changes in neuronal differentiation. As evidence of this, mutations in the genes encoding for cohesin components and MeCP2, responsible for Cornelia de Lange syndrome and Rett syndrome, respectively, are associated with mental development delays [91,92]. Furthermore, patients affected by most of the so-called “chromatinopathies”, Mendelian disorders due to genetic alterations of several components of the epigenetic machinery and chromatin remodellers, display intellectual and neurological dysfunctions, demonstrating how chromatin remodelling plays an essential role in central nervous system development and function [93]. Indeed, high-resolution technologies have recently disclosed that changes in genome 3D organization trace the development of the nervous system by remodulating both the interactions between large chromatin compartments and also the local interactions (i.e., between promoters and enhancers). During neuronal differentiation, a loss of cell stemness is usually associated with the repositioning of key pluripotency-related genes, to repressed chromatin. Differentiation genes are instead characterized by loosened chromatin and are relocated to the active chromatin in the transcriptional factories [94]. Finally, the existence of TADs and chromosomes’ territories, whose positions inside the nucleus could change based on cell type, shows how DNA 3D organization is a common mechanism of DNA regulation that is not restricted to cell differentiation and development. In this regard, the nuclear lamina plays a key role given its ability to bind heterochromatin, which allows it to act as a regulator or stabilizer of DNA organization inside the nucleus. As a result, the alteration of lamins induces several neuronal defects, such as migration problems and nuclear aberrations that lead to dysfunctions in the nervous system. The molecular characteristics and the regulation of the close relationship between chromatin domains/compartments and the nuclear lamina, as well as every element establishing the nuclear architecture, should be investigated in future studies to clarify our knowledge of the neural differentiation process. In addition, some genomic loci that have been associated with neuropsychiatric disorders might be considered in this light, by studying their sensitivity to changes in chromatin architecture. Hence, understanding chromatin–chromatin interactions and chromatin interactions with the nuclear envelope that are able to regulate gene expression programs could be useful in the context of diseases of the nervous system, with the aim of designing and developing new therapeutic strategies. Pathological interactions that lead to specific gene misregulation, indeed, could be considered as novel drug targets. This type of approach has been exploited, for example, in the context of Alzheimer’s disease, where the deletion of an enhancer allowed the ablation of its target gene [95]. The evaluation of novel therapies could also take advantage of the generation of brain organoid models in combination with genome-wide approaches. 

The focus of this review is neuronal development. However, it will also be of interest to evaluate the changes in the 3D organization of chromatin and how these changes in chromatin arrangement are orchestrated during neuronal maturation, particularly when neurons acquire their specific morphology and, thus, the electrophysiological and molecular characteristics that allow neuronal function in the central nervous system. Additional data would also be needed in order to confirm, in other contexts of cell differentiation, and refine this scenario of chromatin architecture and nuclear lamina collaboration in cell differentiation. This can provide insights into the process of cell differentiation and, more widely, of DNA regulation. 

## Data Availability

Not applicable.
